# Surgery Treatment of Primary Tumors of the Inferior Vena Cava

**DOI:** 10.3389/fmed.2022.770967

**Published:** 2022-03-02

**Authors:** Shizhi Wang, Yuqiu Li, Qijun Yang, Xue Zhang, Yunqi Cheng, Zimeng Li, Jingyi Wang, Qingfu Zeng

**Affiliations:** ^1^Department of Vascular Surgery, The Second Affiliated Hospital of Nanchang University, Nanchang, China; ^2^Queen Mary College of Nanchang University, Nanchang, China; ^3^School of Public Health, Nanchang University, Nanchang, China

**Keywords:** intravenous leiomyomatosis, inferior vena cava leiomyosarcoma, surgical treatment, prognosis, inferior vena cava

## Abstract

**Background:**

Primary tumor of the inferior vena cava is a rare tumor, which arises from the smooth muscle of vascular walls. Surgery appears the only curative treatment. However, the optimal surgical methods and surgical management are not well-studied. In this article, we reviewed the successful treatment experience of patients in our center who had resection of primary tumor of the inferior vena cava and reviewed the relevant literature.

**Methods:**

Four cases of patients who undergoing initial resection of primary tumors of the inferior vena cava from September 2017 to August 2021 in the Second Affiliated Hospital of Nanchang University were screened and followed up. They were discussed and cases reported in this field were reviewed.

**Results:**

Among the four patients, three of them were female. The median age of the disease is 53.75 years (range 45–60 years). After surgical treatment, tumors were removed in all patients, and some patients had reconstruction of inferior vena cava. There were no disease-specific deaths, no serious complications, and no recurrence during follow-up in these cases.

**Conclusions:**

Careful preoperative examination, correct surgical treatment methods, and multidisciplinary collaboration can lead to safe and successful operations, which improve the survival rate of patients.

## Introduction

Primary tumors of the inferior vena cava are rare tumors, most of which are derived from the endothelial smooth muscle of the intima (1). These tumors can be classified as benign or malignant. Benign smooth muscle tumors are called leiomyomatosis and malignant ones are leiomyosarcoma. Since most patients have non-specific complaints, such as abdominal pain and low back pain, the diagnosis of this disease is sometimes delayed, and challenging. It is also because of these atypical symptoms and high incidence among female compared with male that the receiving physicians may first associate with gynecological disease, rather than vascular disease, which is against making correct diagnosis decisions. Techniques such as computed tomography venography (CTV), magnetic resonance venography (MRV), and intraoperative digital subtraction angiography (DSA) are useful diagnostic methods (2). Histopathology and immunohistochemistry examination assessed by pathologist are recommended to provide further evidences of the nature of the tumor and develop appropriate treatment plans postoperatively if necessary.

At present, the main and most effective treatment for this disease is surgical Resection with clear margins. Complete resection is necessary since it is strongly associated with the prognosis and metastasis risks (3). However, the adjacent structures of the inferior vena cava are complex, and tumors often grow into the right atrium or even into the pulmonary artery, which bring tough challenges to clinical treatment. Furthermore, the management after the surgery resection of tumors is also vital. It is still controversial for the need and different strategies used for inferior vena cava reconstruction, which depends on the tumor location and hepatic or renal vein involvement (4). However, in recent years, there are few clinical studies and reference experience on leiomyoma. Therefore, we present four patients who had primary tumors of the inferior vena cava and been successfully treated in our center, and summarize the experience of diagnosis and treatment of this disease in combination with the literature, which may help clinicians who do not know this rare disease to differential diagnose leiomyomatosis and leiomyosarcomas of inferior vena cava and increase curative ratio of this disease.

## Materials and Methods

In this article, we reported four typical patients undergoing initial resection of primary tumors of the inferior vena cava from May 2020 to August 2021 in the Second Affiliated Hospital of Nanchang University.

Relevant medical records were used to assess the symptoms and assist the diagnosis, treatment and prognosis. All preoperative examinations were used to show the patient's physical condition, as well as the size, location, and source of the tumor, so that a reasonable surgical plan could be proposed. Surgical reports recorded the surgical procedure and details of the patient's tumor. Pathological reports provided strong support for the judgment of tumor grade and tumor nature. Follow-up clinical records were used to record the postoperative recovery of patients, which were convincing ways to assess the outcome of surgery in a certain extent. We continued to track the patient's health status and survival time from the time of surgery.

In addition, in order to make a more comprehensive introduction of the disease and conduct a comprehensive research of all reported cases, we also searched the Cochrane Library, Pubmed, MEDLINE, EMBASE, Elsevier, and Springerlink databases with the words “leiomyosarcoma, leiomyoma, or inferior vena cava tumor.” The search results demonstrated symptoms, diagnostic methods, and different surgical treatments. Combined with our understanding and experience in the diagnosis and operative treatment of this disease, this article may provide references for better treatment of the disease in the future.

## Results

### Case 1

**Figure 1:**
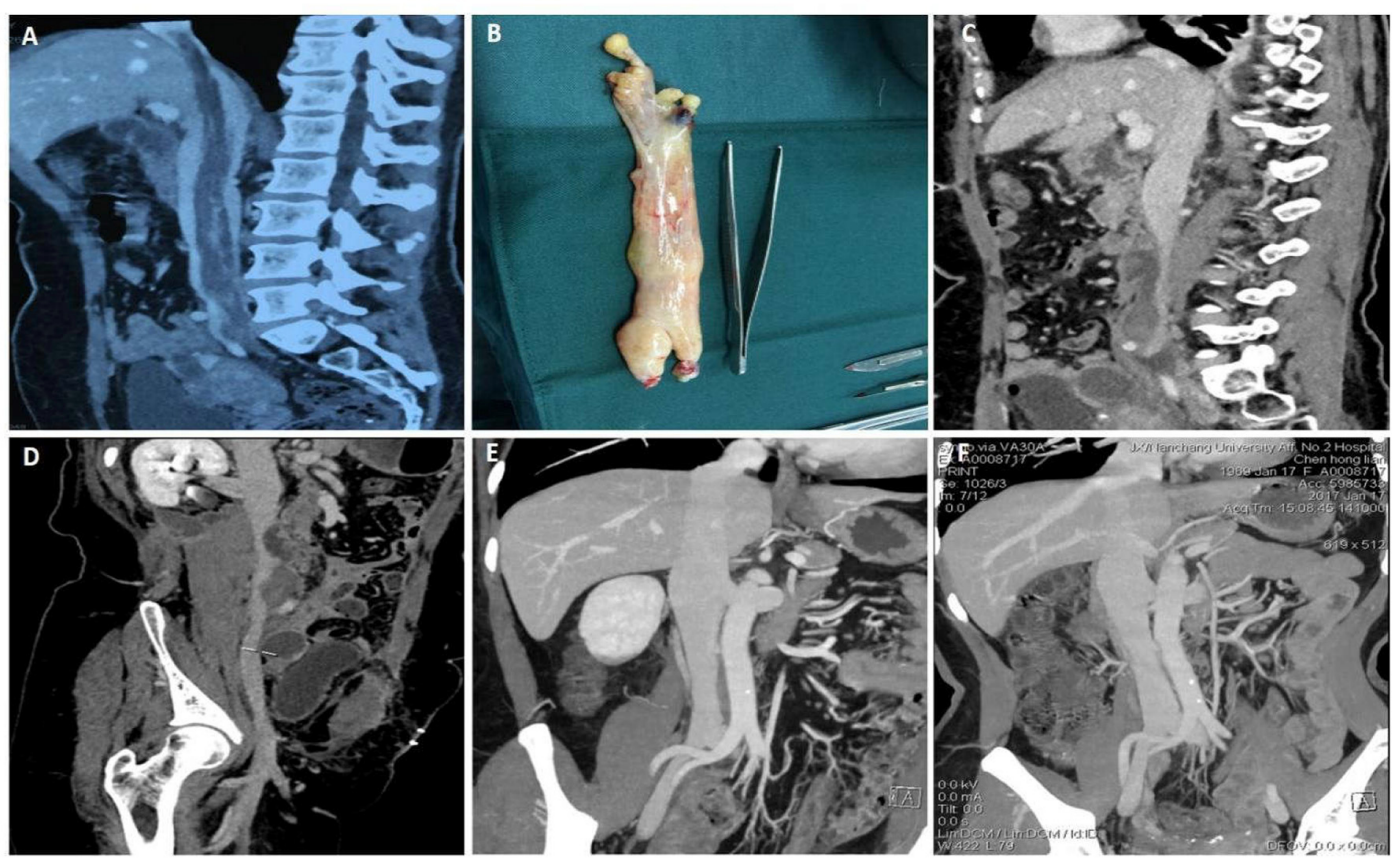
Data of case 1 before, during, and during follow-up. **(A)** Preoperative computed tomography venography (CTV) confirmed a mass that extended from the right internal iliac vein to the proximal inferior vena cava and extended into the right atrium. **(B)** An intact yellow mass was removed from the right atrium and proximal to the inferior vena cava. **(C,D)** Seven days after surgery, CTV confirmed that the mass was removed and the inferior vena cava was unobstructed. **(E,F)** Six-month follow-up CTV and 12-month follow-up CTV revealed no residual mass or recurrence and the prognosis was good.

A 45-year-old female was admitted to hospital with abdominal distension of 3 months. She had undergone open hysterectomy and adnexectomy 3 months before. Preoperative CTV suggested a space occupying lesion of inferior vena cava, which extended from the right internal iliac vein to the proximal inferior vena cava and finally extended into the right atrium (Figure 1A). After laparotomy, we dissected the inferior segment of the inferior vena cava and cut the anterior wall. A soft yellow tumor was found without adhesion to the wall of the inferior vena cava (Figure 1B). During the operation, the tumor in the right atrium and the proximal end of the inferior vena cava was carefully removed completely. Pathological examination suggested that the tumor was a leyomioma (Figures 1C,D). Seven days after the surgery, CTV revealed the tumor was removed and the inferior vena cava was patent. The patient recovered well and was discharged 8 days after the surgery. Six months after the operation, reexamination of CTV showed no tumor residue or recurrence. Follow-up CTV 2 years after the operation confirmed a good prognosis (Figures 1E,F).

### Case 2

**Figure 2:**
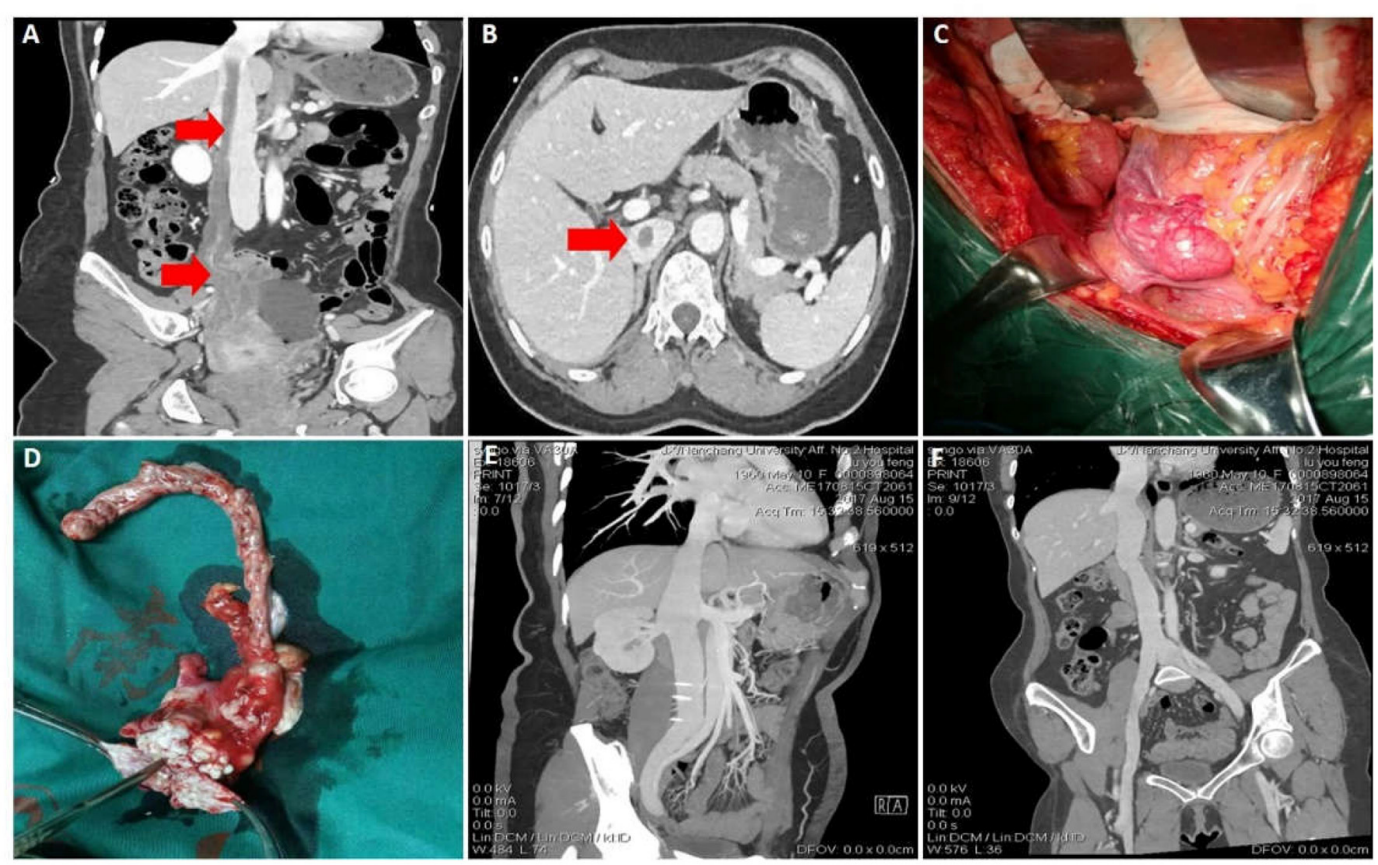
Data of case 2 before, during, and during follow-up. **(A,B)** Preoperative CTV and abdominal CT scan showed a mass extended through the right ovarian vein to the huge occupying lesion in the inferior vena cava. **(C)** Inferior vena cava tumor and right ovarian vein were removed, and the right adnexa and part of uterus were also removed. **(D)** The intact mass was removed. **(E,F)** Eight months after surgery, CTV confirmed that the inferior vena cava was unobstructed and the prognosis was good.

A 52-year-old woman presented to hospital with lower abdominal pain for 1 month. and worsening for half a month. She had uterine fibroid surgery 5 years before. Preoperative CTV and gynecological color ultrasound suggested that the right adnexa area and the bottom of the uterus were occupied by a mass. The lesion extended through the right ovarian vein to the huge occupying lesion in the inferior vena cava, and the mass reached the entrance of the right atrium (Figures 2A,B). In this case, a multidisciplinary operation was necessary and useful. Gynecologists excised right adnexa and part of uterus. Vascular surgeon excised the mass of inferior vena cava and right ovarian vein (Figures 2C,D). Histopathological examination of the surgical specimen suggested intravenous leiomyomatosis but no tumor in the fallopian tube. Microscopically, in the right adnexal and parauterine tissues, the tumor cells were spindle shaped, growing around the blood vessels, and the atypia was slight. Besides, IHC study revealed positivity for smooth muscle actin and desmin and Ki-67 positivity <5% of tumor cells and was consistent with the diagnosis of leiomyomatosis. Eight months after the operation, the patient rechecked the CTV of inferior vena cava and no tumor residue or recurrence was found (Figures 2E,F). The patient recovered well without serious complications and still rechecked the CTV of inferior vena cava regularly.

### Case 3

**Figure 3:**
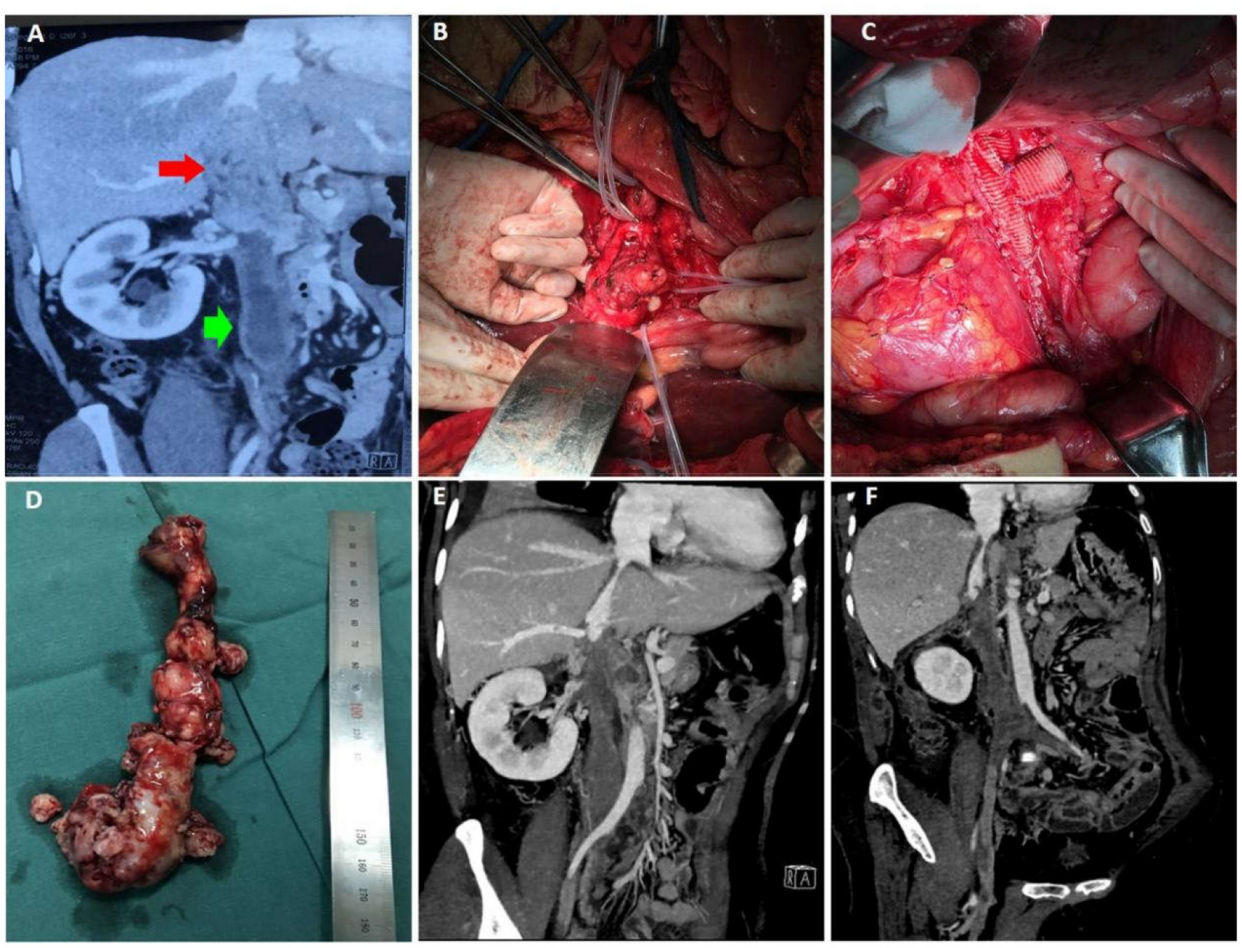
Data of case 3 before, during, and during follow-up. **(A)** Preoperative CTV suggested a space-occupying lesion of the inferior vena cava, which started at the level of the junction of the hepatic vein and elongated to the junction of the renal vein. **(B,C)** During the operation, the liver was dissociated and the first and second hilum were controlled with tourniquets. After complete resection of the inferior vena cava mass, artificial vascular patches were used to reconstruct the inferior vena cava. **(D)** The intact mass was removed. **(E,F)** Ten days after surgery, CTV confirmed that the inferior vena cava was unobstructed and the prognosis was good.

A 60-year-old female presented to hospital with a 6-month history of edema of both lower limbs and the discovery of mass in the inferior vena cava for 1 month. Preoperative CTV suggested a space-occupying lesion of the inferior vena cava. The upper pole of the mass was at the level of the junction of the hepatic vein and the lower pole at the junction of the renal vein (Figure 3A). The mass caused the formation of the thrombi in the renal vein as well as in the distal inferior vena cava, left renal vein, and both common iliac veins. Moreover, the thrombi occluded the lumen of these veins completely. During the operation, with the assistance of hepatobiliary surgery, a median abdominal longitudinal incision combined with a right upper abdominal transverse incision were performed to completely dissociate the liver, and the first and second hila hepatis were controlled by tourniquets to temporarily close the blood vessels. After complete liver dissociation, the inferior vena cava mass was completely excised. To reconstruct the inferior vena cava, artificial vascular patches were used and left renal vena-inferior vena cava grafts were performed (Figures 3B–D). The operation went well and pathological examination suggested leiomyosarcoma.

The patient was taken to the intensive care unit after the operation. Ten days after the surgery, CTV revealed no tumor residence and recurrence (Figures 3E,F). She was allowed to go home 14 days after the surgery in good condition. Now, she continues to have no evidence of recurrence at the time of this report, 12 months after her initial operation.

### Case 4

**Figure 4:**
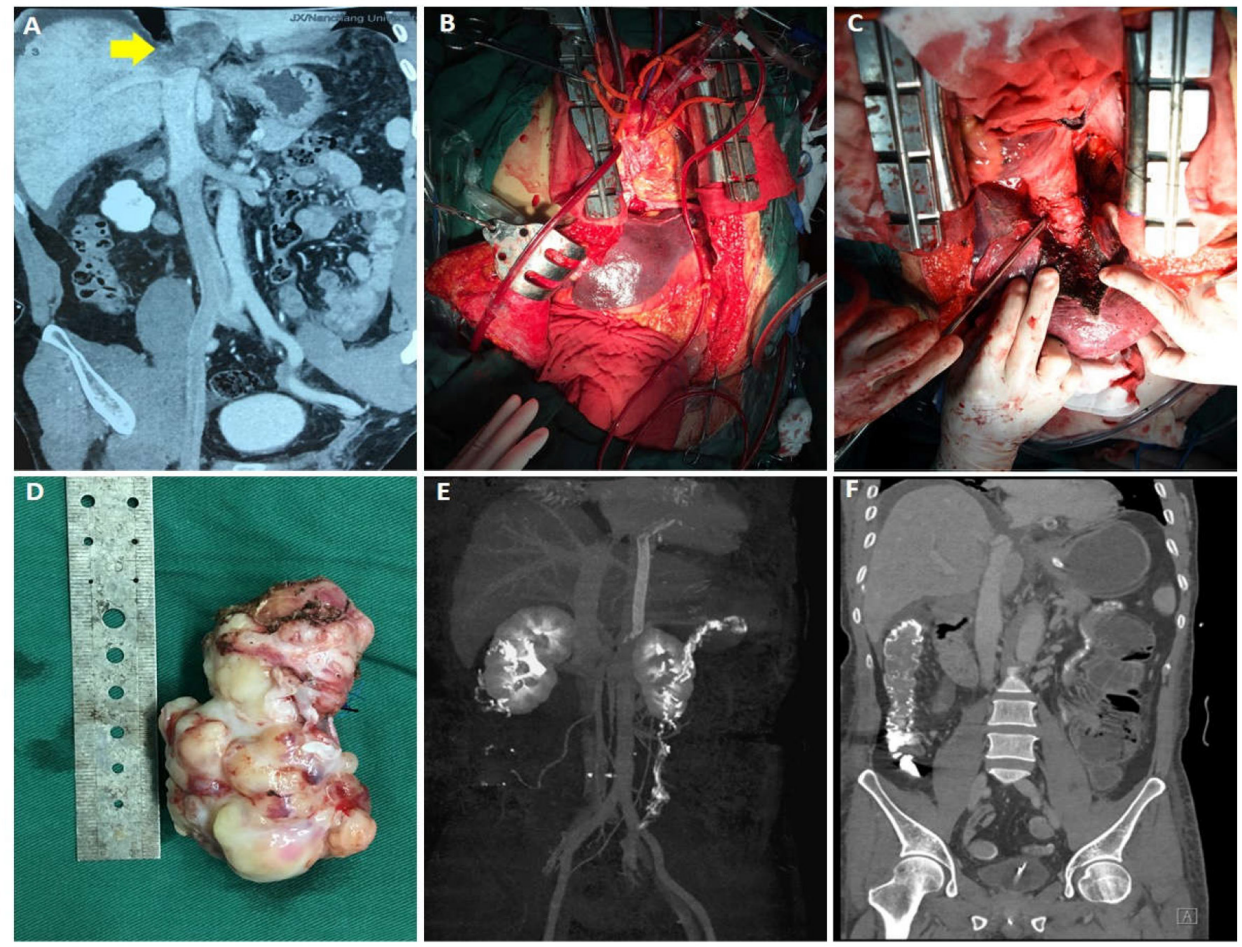
Data of case 4 before, during, and during follow-up. **(A)** Preoperative CTV revealed that a mass invaded the second hilum and involved the hepatic vein. **(B,C)** During the operation, the liver was completely dissociated and the first hilum was controlled. The right atrium was cut open to separate the intraatrial mass, and then the mass at the second hilum was gradually dissociated. **(D)** An intact mass was removed. **(E,F)** Thirteen-day follow-up CTV revealed no residual mass or recurrence and the prognosis was good.

A 58-year-old man with upper abdominal pain for 1 month presented to his hospital. Preoperative CTV and MRI indicated a large mass lesion in the inferior vena cava. The mass invaded the second hepatic portal, obstructed the hepatic vein, extended into the right atrium, and caused complete obstruction of the right atrial portal, which was considered as secondary Budd-Chiari syndrome caused by the mass (Figure 4A). The surgical plan for him was developed by a multidisciplinary collaboration of vascular, cardiac, and hepatobiliary surgery. After cardiac arrest under extracorporeal circulation, a combined thoraco-abdominal incision was used to completely free the liver and control the first hepatic hilum. The right atrium was dissected to separate the mass in the atrium. Then the mass at the second hepatic hilum was gradually dissociated (Figures 4B,C). Finally, the tumor was removed (Figure 4D). The defect in the inferior vena cava was reconstructed with bovine pericardial patch. The pathological examination demonstrated leiomyosarcoma, which may require subsequent adjuvant radiotherapy. The patient recovered well without serious complications. Thirteen days after the operation, CTV revealed the good prognosis (Figures 4E,F). Fifteen days after the operation, he was discharged from hospital in general good condition.

## Discussion

In our study, we present a series of four cases with primary tumors of inferior vena cava which underwent surgery resection. Summarizing the four patients, we found that three of them were female. The first two patients had leiomyomatosis while the last two patients had leiomyosarcoma (Table 1).

**Table 1:** Overview of cases.

**Patient**	**Patient** **age**	**Gender**	**Symptom**	**Relevant medical** **history**	**Tumor type**	**Type of** **IVC repair**
1	45	Female	Abdominal distension	Open hysterectomy and adnexectomy	Leiomyomatosis	Ligation
2	52	Female	Lower abdominal pain	Uterine fibroid surgery	Leiomyomatosis	Ligation
3	60	Female	edema of both lower limbs	No	Leiomyosarcoma	Artificial vascular patch and left renal vena-inferior vena cava graft
4	58	Male	Upper abdominal pain	No	Leiomyosarcoma	Bovine pericardial patch

It is known that the incidence of IVL is low, which may also be related to missed diagnosis and misdiagnosis. The first IVL case was reported by Birch-Hirschfeld in 1896 (5). Composed of nodular masses of smooth muscle cells, IVL is often found within venous vascular spaces of the uterine wall and benign, which are the common histological features. However, some tumors have malignant biological behavior, which may grow invasively and even infiltrate blood vessel walls (6, 7). Because the tumor grows into the uterine vein and then can affect the iliac vein, inferior vena cava, right atrium, and even the pulmonary artery, IVL can directly cause different degrees of vascular occlusion and high mortality. Heart involvement rate of IVL can be as high as 10–30% (8, 9). The pathogenesis of IVL is unclear, but it may be due to invasion of leiomyoma into the uterus and parauterine veins or the smooth muscle cells in the vascular wall growing into the lumen (10). There are several vital factors that contributed to the occurrence of IVL, such as elevated estrogen levels, venous blood stasis, and local damage (11). Cesarean section and intrauterine device placement may also be associated with IVL (12).

Inferior vena cava leiomyosarcoma (IVCL) was first reported by Perl in 1871 (13). So far, only about 400 cases have been reported in the literature. From the reported literature, the mean age of diagnosis was 55 years (range 45–63 years). Although the biological mechanism of gender predilection has not been elucidated, there is a significant female predominance. The ratio of women to men was 3.25:1 (14).

According to the meta-analysis of Watchtel et al., the most common presenting symptoms for IVCL patients were abdominal pain (60%), lower extremity edema (15%), and weight loss (11%) (14). Other symptoms such as abdominal distention, deep venous thrombosis, and palpable mass may also be present. About half of the tumors were located in the inferior vena cava segment between hepatic vein and renal vein. Six percent of the tumors entered the right atrium, and 60% of the tumors protruded outside the lumen (14). As for our patient, worsening chronic abdominal pain is the most common symptom in them, which is consistent with the results reported in the literature. However, in our four patients, three of them had tumors extending into the right atrium, which seems to be higher than 60%. It may be because the patient did not pay attention to the symptoms such as abdominal pain before, resulting in a long course of disease and a long time for tumors to grow and enter the right atrium. All of our patient underwent surgery, while two patients underwent inferior vena cava reconstruction, one with bovine pericardial patch, the other with artificial vascular patches and left renal vena-inferior vena cava grafts. All of them recovered well-postoperatively.

The pathogenesis of IVCL has not been elucidated, but it may be related to the abnormal function of endocrine system. Estrogen may play an important role in the development of this disease. Most cases occur in women around menopause and estrogen receptors on tumor smooth muscle membranes are increased. Three of our four patients were also women, which seems to be consistent with this hypothesis. However, given the small sample size, we need to further enroll more patients and perform immunohistochemical tests in the future to verify this hypothesis.

The clinical diagnosis of the primary tumor of inferior vena cava is difficult because the symptoms are not-specific. Multiple imaging techniques play an important role in preoperative diagnosis. Some patients have no symptoms, but they are discovered by chance through physical examination, color ultrasound, or imaging examination of other diseases. Besides, imaging techniques can also be used to make an effective preliminary judgment of the nature and source of the tumor. But there is no uniform standard for histological identification of benign and malignant smooth muscle tumors. Generally, according to the mitotic phase as the most important criterion, the absence of mitotic phase is benign.

The most effective treatment for the primary tumor of inferior vena cava is surgery. For IVL, in principle, removal of the uterus, even the adnexa of the uterus, is necessary for most patients. The gold standard of surgical treatment for IVL is hysterectomy and bilateral salpingectomy proposed by many studies. Hysterectomy is suitable for most patients. Bilateral oophorectomy may be recommended only for postmenopausal or near-menopausal patients or patients who have extrauterine vascular infiltration. Myomectomy is only recommended for young women who have fertility needs (8). Whatever surgical methods we use, completing resection of the tumor is essential to obtain a positive prognosis regarding remission and recurrence. However, in recent years, series of studies suggest that treatment of IVL with gonadotropin-releasing hormone agonists after incomplete resection is as effective as complete resection. Antiestrogen therapy has also been recommended to prevent tumor growth and reduce recurrence rate (15). However, it is reported that the tumor adhered to the vascular wall, leading to laceration of the inferior vena cava and massive bleeding during surgery (16).

For IVCL, because tumors often invade surrounding structures and organs, most of the surgery is performed in combination with multi-disciplinary procedures. Combined organ resection was common, such as right kidney resection (64.9%), right adrenal gland resection (27.3%), partial liver resection (20%), and abdominal aorta resection (6.1%). Extracorporeal circulation was used in 6.4% of cases to assist to excise the mass protruding into the heart (14). Ligation, patch repair, or graft replacement are possible management of the inferior vena cava. According to the experience of our center, multidisciplinary collaborative surgery is more suitable and may be assisted by vascular surgery, cardiac surgery, gynecology, and hepatobiliary surgery to resect tumors as completely as possible. And functional reconstruct the inferior vena cava is also a good way to achieve the best patient outcomes (17).

The location of the tumor and the degree of local invasion make the surgical approach different. In relation to hepatic and renal veins, IVCL can be divided into three segments anatomically. Lower segment refers below the renal vein to the bifurcation of the inferior vena cava, which is the most easily excised site for IVCL. Middle segment runs from the hepatic veins to the renal veins. To fully expose, the middle segment resection of IVCL should be performed through a combined thoracoabdominal incision. The key of this kind of operation is how to ensure the bilateral renal vein reflux after resecting the middle IVC including the left and right renal vein entrances. Upper segment is from the entrance of the hepatic vein to the right atrium (18). Tumors in this segment can lead to hepatic vein occlusion, which cause the Budd-Chiari syndrome. There is no acceptable surgical treatment for tumors in this segment (19).

Although IVCL can be removed surgically, this does not guarantee a long-term survival for patients. In most patients, the primary tumor will eventually metastasize. Local recurrence rate is 33% of the patients, and distal recurrence rate is 48% (20). Five-year survival of patients was 55%. The reasons for the low 5-year survival rate are multifactorial. Age more than 55 years old, tumor volume >9 cm, positive surgical margin, and tumor location in the upper hepatic vein or lower renal vein are all important factors (14). Patients with the best long-term survival have radical surgical resection with a negative margin and no metastasis at the time of surgery (21). In our study, patients were treated only with surgery, and during nearly 3 years of follow-up, they did not relapse or die, probably because their tumors were less aggressive and they were completely removed. Hollenbeck et al. found that 3-year survival of patients with negative margins was 76% compared with 0% in patients with positive margins (22). Radiation and chemotherapy are often used, but the effects are unclear. Some scholars believe that adjuvant chemotherapy is useful and can improve local effects. The preoperative external-beam radiations seems to facilitate marginally negative resection (23). However, some studies also showed the adverse effects about neoadjuvant or adjuvant chemotherapy (24, 25). This means that the efficacy of chemotherapy and is still controversial.

## Conclusion

In this study, we present four cases of primary tumor of the inferior vena cava. These cases were effectively treated and had a relatively good prognosis. Combined with the research review, we summarized the experience of the symptoms, diagnosis, treatment, and prognosis of this disease. Imaging examination is essential for diagnosis of primary tumors of the inferior vena cava. Surgical resection is the curative treatment for the disease, especially multidisciplinary collaborative surgery, which may be assisted by experienced vascular, gynecological, and cardiac surgeons. Perioperative planning, appropriate intraoperative surgical methods, coordination, and postoperative timely examination are necessary for treatment of this disease.

## Data Availability Statement

The original contributions presented in the study are included in the article/supplementary material, further inquiries can be directed to the corresponding author/s.

## Ethics Statement

The studies involving human participants were reviewed and approved by the Medical Ethics Committee of the Second Affiliated Hospital of Nanchang University. The patients/participants provided their written informed consent to participate in this study. Written informed consent was obtained from the individual(s) for the publication of any potentially identifiable images or data included in this article.

## Author Contributions

SW did the acquisition of data. YL carried out writing the manuscript and searching for references. QY and XZ searched for references and revised the manuscript. YC, ZL, and JW did the revision. QZ did the study design and final revision. All authors contributed to the article and approved the submitted version.

## Funding

This study received funding from the Jiangxi Provincial Health Commission Foundation (No. SKJP-220211495).

## Conflict of Interest

The authors declare that the research was conducted in the absence of any commercial or financial relationships that could be construed as a potential conflict of interest.

## Publisher's Note

All claims expressed in this article are solely those of the authors and do not necessarily represent those of their affiliated organizations, or those of the publisher, the editors and the reviewers. Any product that may be evaluated in this article, or claim that may be made by its manufacturer, is not guaranteed or endorsed by the publisher.
